# Atmospheric Heavy Metal Pollution Characteristics and Health Risk Assessment Across Various Type of Cities in China

**DOI:** 10.3390/toxics13030220

**Published:** 2025-03-17

**Authors:** Zhichun Cha, Xi Zhang, Kai Zhang, Guanhua Zhou, Jian Gao, Sichu Sun, Yuanguan Gao, Haiyan Liu

**Affiliations:** 1 State Key Laboratory of Environmental Criteria and Risk Assessment, Chinese Research Academy of Environmental Sciences, Beijing 100012, China; chazhichun0509@163.com (Z.C.); x-zhang@kitakyu-u.ac.jp (X.Z.); scsun1117@163.com (S.S.); gaoyg@craes.org.cn (Y.G.); 2 Faculty of Environmental Engineering, The University of Kitakyushu, 1–1 Hibikino, Wakamatsu, Kitakyushu 808-0135, Fukuoka, Japan; 3 School of Instrumentation and Optoelectronic Engineering, Beihang University, Beijing 100191, China; zhouguanhua@buaa.edu.cn; 4 Yinchuan Ecological Environment Monitoring Centre, Yinchuan 640100, China; 15804665746@163.com

**Keywords:** PM_2.5_, resource-industrial cities (RICs), general cities (GCs), atmospheric heavy metal pollution, health risk assessment

## Abstract

This study investigates the spatiotemporal trends and health risks of nine atmospheric heavy metals (Pb, As, Mn, Ni, Cr, Cd, Zn, Cu, Fe) in PM_2.5_ across 50 Chinse cities, comparing resource-industrial cities (RICs) and general cities (GCs) before (2014–2018) and after (2019–2021) China’s 2018 Air Pollution Prevention and Control Action Plan. Post-2018, concentrations of all metals except Fe declined significantly (33–77%), surpassing PM_2.5_ reductions (25%). Geospatial analysis revealed elevated heavy metal levels in northern and southern regions in China, aligning with industrial and mining hotspots. While RICs exhibited persistently higher metal concentrations than GCs, the inter-city gap narrowed post-2018, with RICs achieving greater reduction. Pre-2018, the combined non-carcinogenic hazard index (HI < 1) remained below safety thresholds, but the combined carcinogenic risk total (CRT) for children exceeded 10^−4^, driven primarily by As and Cr(VI). HIs were 1.5–2.0 times higher in RICs than in GCs. Post-2018, the CRT declined by 69.0–71.1%, aligning with reduced heavy metal levels. Despite improvements, CRTs necessitate targeted mitigation for As (contributing 81.1–86.2% to CRT) and Cr(VI) (11.7–14.0%). These findings validate the policy’s effectiveness in curbing industrial and vehicular emissions but underscore the need for metal-specific controls in resource-intensive regions to safeguard child health.

## 1. Introduction

In recent years, atmospheric particulate matter, particularly PM_2.5_, has emerged as a pollutant in China’s ambient air, linked to significant public health risks, including respiratory and cardiovascular diseases [1,2,3]. Globally, particulate matter pollution accounts for 3% of deaths from cardiopulmonary diseases and 5% of lung cancer fatalities [4,5]. Heavy metals, including arsenic (As), cadmium (Cd), chromium (Cr), copper (Cu), manganese (Mn), nickel (Ni), lead (Pb), and zinc (Zn), are critical components of PM_2.5_ that can induce various toxic effects through inhalation [6]. Metals like Mn and Ni can catalyze hydroxyl radical production, inducing cellular damage through lipid peroxidation and protein oxidation [7], while Zn, Cr, Cu, and Pb exhibit synergistic toxicity in lung epithelial cells [8]. Prenatal exposure to As, Cd, Mn, and Pb has been linked to childhood asthma [9]. The World Health Organization (WHO) identifies As, Ni, Cr(VI), Cd, and other heavy metals as carcinogens, establishing stringent air quality guidelines to mitigate exposure [10].

Atmospheric heavy metals primarily originate from industrial emissions, mining activities, vehicular exhaust, fossil fuel combustion, waste incineration, construction, and crustal dust [11,12]. Urban pollution profiles vary significantly due to the differences in energy structures and industrial composition, [13]. Current studies predominantly focus on specific cities or regions. For instance, Pb and Cu levels in urban and traffic areas of Navarra, Spain, significantly exceed the rural background, although remaining below WHO thresholds [14]. Similarly, petrochemical operations emit substantial Pb, Hg, Ni, and Cr in the surrounding environment [15], while coal mining areas show the dominance of Zn, Mn, and Pb, constituting over 82% of total metal content [16]. In China, a meta-analysis of 14 cities identified Foshan, Wuhan, Xi’an, Jinan, and Shenzhen as hotspots for Pb, As, Ni, Cr, and Cd pollution [17]. Regional studies have further characterized heavy metals trends: Duan et al. analyzed pre-2013 pollution patterns in Chin [18], while Li et al. revealed a south to north enrichment gradual linked industrial structure distribution [19]. Yu et al. noted declining PM_2.5_ -bound metals (Cd, Cr, Ni, Pb, Zn, Hg, and As), except for the element Cu, since 2017 [20].

Despite these insights, systematic comparisons of heavy metal pollution between resource-industrial cities (RICs) and general cities (GCs) remain limited, particularly in assessing how industrial emissions, vehicle density, and coal combustion drive spatial disparities. Mining and smelting activities—such as crushing, grinding, and refining—generate particulate matter laden with hazardous metal concentrations [21], while industries like steel manufacturing and cement production exacerbate air quality deterioration [22]. Existing studies often sample in mixed-use areas of residential, commercial, and transportation to mitigate the influence of dominant industrial sources. However, this approach may introduce errors if classification cities rely solely on the national list of resource cities (2013), thus compromising the accuracy and representativeness of the results.

The Three-Year Action Plan to Win the Blue Sky Defense War (2018–2020) launched by China’s Central Government prioritized regional air quality governance. This policy calls for vigorously adjusting and optimizing the industrial structure, energy structure, transportation structure, and land use structure and strengthening regional joint prevention and control. Comprehensively treating industrial pollution, building a clean, low-carbon, and efficient energy system, and implementing major special actions significantly reduce pollutant emissions. To address this, local governments enhanced their efforts to understand and control heavy air pollution and tightened the prevention and control measures of heavy air pollution, leading to a significant reduction in atmospheric heavy metals pollution levels across various cities [20].

This study addresses these gaps by analyzing PM_2.5_-bound heavy metal (Pb, As, Mn, Ni, Cr, Cd, Zn, Cu, Fe) in 50 Chinese cities pre- (2014–2018) and post-policy (2019–2021). We categorized the collected cities into resource-industrial cities (RICs) and general cities (GCs) based on functional zoning and sampling site representativeness, avoiding biases from oversimplified classifications. The primary objective is to (1) compare the spatiotemporal distribution changes in PM_2.5_ and its associated heavy metal concentrations before and after the 2018 policy implementation. Additionally, it analyzed the differences in PM_2.5_ and heavy metal concentrations between the RICs and GCs areas. Aecondary objectives are to (2) quantify health risks (combined non-carcinogenic hazard index, HI; combined carcinogenic risk total, CRT) for adults and children and (3) evaluate the efficacy of the 2018 policy in mitigating disparities and residual risks. These findings may provide policymakers with actionable strategies for refining emission controls in high-risk regions and safeguarding public health.

## 2. Materials and Methods

### 2.1. Data Sources and Processing

The data were obtained from the literature from 2014 to 2021, as well as from existing national air quality monitoring data. The literature inclusion followed strict criteria about data quality, temporal coverage, and analytical methods: (1) Studies must report the PM_2.5-_bound heavy metals concentration (Pb, As, Mn, Ni, Cr, Cd, Zn, Cu, and Fe) with a documented quality control procedure; (2) The sampling period was between 2014 and 2021, with ≥20 samples collected cumulatively and the sampling period exceeding one month (>30 days); (3) Analytical methods for concentration quantification via inductively Coupled Plasma Mass Spectrometry (ICP-MS), Inductively Coupled Plasma Emission Spectrometry (ICP-AES), or X-ray fluorescence (XRF). A total of 63 datasets spanning 50 cities met these criteria. The geometric means and standard deviations of PM_2.5_ and heavy metals concentrations for each city were used to account for log-normal distribution patterns in environmental data.

### 2.2. Methodology for Classifying Urban Typologies

Cities are classified as RICs and GCs were based on the National Sustainable Development Plan for Resource Cities (2013–2020) and the functional zoning of sampling sites. RICs were defined as cities where natural resources extraction (e.g., minerals and forestry) dominants the economy, with sampling sites located in or near industrial zones (e.g., smelters, coral-fired plants). GCs included urban areas with sampling sites in residential or commercial zones, lacking proximate industrial activity. For example, cities with sampling points within industrial areas were categorized as RICs, while others were designated GCs. This dual classification—combining policy definitions with a spatial context—ensures the representative characterization of industrial emission impacts on atmospheric heavy metal concentrations.

### 2.3. Health Risk Assessment

This study assessed health risk associated with PM_2.5_-bound heavy metal using the U.S. Environmental Protection Agency (EPA) risk assessment model [23,24,25]. The participants were categorized into adult and child to account for differences in physical characteristics and respiratory systems. Non-carcinogenic risk (hazard quotient, HQ) and carcinogenic risk (CR) were evaluated across three exposure pathways: ingestion, inhalation, and dermal contact. For chromium, only hexavalent chromium (Cr(VI)), calculated as 1/7 of the total Cr concentration, was considered a carcinogen [26].

The exposure formulas of the three pathways are as follows [24]:

(1) $${CDI}_{ing}=C_{ing}\times \frac{IngR\times EF\times ED\times CF}{BW\times AT}$$

(2) $${EC}_{inh}=C_{inh}\times \frac{ET\times EF\times ED}{{AT}_{n}}$$

(3) $${DAD}_{der}=C_{der}\times \frac{SA\times AF\times ABS\times EF\times ED\times CF}{BW\times AT}$$

where *CDI_ing_* is the daily intake via ingestion (mg·(kg·d)^−1^); *EC_inh_* is the inhalation exposure concentration (μg·m^−3^); *DAD_der_* is the dermal absorbed dose (mg·(kg·d)^−1^); *C**_inh_*** is the concentration of heavy metals in particulate matter (μg·m^−3^); and *C**_ing_*** and *C**_der_*** are the contents of heavy metals in particulate matter (mg·kg^−1^). The definitions and values of other parameters in the formulas are shown in Table 1.

Equations (4)–(9) were used to calculate the HQ and CR of a single element to the human body:

(4) $${HQ}_{ing}=\frac{{CDI}_{ing}}{{RfD}_{0}}\;\;\;\;$$

(5) $${HQ}_{inh}=\frac{{EC}_{inh}}{{RfC}_{i}\times 1000}\;\;\;\;\;\;\;\;\;$$

(6) $${HQ}_{der}=\frac{{DAD}_{der}}{{RfD}_{0}\times GIABS}\;\;\;$$

(7) $${CR}_{ing}={CDI}_{ing}\times {SF}_{0}$$

(8) $${CR}_{inh}={EC}_{inh}\times IUR$$

(9) $${CR}_{der}={DAD}_{der}\times \frac{{SF}_{0}}{GIABS}$$

To assess the overall potential for non-carcinogenic risks and carcinogenic risks posed by multi-element exposure, the combined non-carcinogenic HI and combined CRT were estimated as the sum of *HQi* and *CRi*, assuming additive effects [23,24,27]. The HI and CRT are calculated as follows:

(10) $$HI=\sum {HQ}_{i}\;$$

(11) $$CRT=\sum {CR}_{i}$$

In the formulas, RfD_0_, RfCi, GIABS, SF_0_, and IUR represent the oral reference dose, inhalation reference concentration, gastrointestinal absorption factor, oral slope factor, and inhalation unit risk, respectively. The parameter values are detailed in Table 2 [24]. When HI ≤ 1, there is no non-carcinogenic risk; when HI > 1, a non-carcinogenic risk is indicated. When CRT < 10^−6^, there is a negligible risk; when 10^−6^ ≤ CRT ≤ 10^−4^, there is a tolerable risk; and if CRT > 10^−4^, there is a significant cancer risk.

## 3. Results and Discussion

### 3.1. Changes in PM_2.5_ and Heavy Metal Concentrations Before and After 2018

The specific content and proportions of heavy metal elements in PM_2.5_ across cities and regions in China before and after 2018 are detailed in Table A1. The average concentration of PM_2.5_ decreased by 25%, from 76.3 μg·m^−3^ before 2018 to 57.3 μg·m^−3^ after 2018. This decline indicates the effectiveness of the Three-Year Action Plan to Win the Blue Sky Defense War promulgated and implemented after 2018. This policy-driven improvement aligns with increased air quality compliance rates and reduced heavily polluted days post-2018 [28].

Pre-2018, the dominant heavy metal in PM_2.5_ were Fe (741.2 ng·m^−3^), Zn (434.7 ng·m^−3^), and Pb (127.0 ng·m^−3^), followed by Cu, Mn, Cr, As, Ni, and Cd (Table A1). Post-2018, all metals except Fe exhibited significant reductions: Cu (−77.1%), Cd (−73.7%), Ni (−73.1%), and Cr (−70.6%) showed the steepest declines, while Fe concentrations decreased marginally (−5.7%), with its proportional contribution to PM_2.5_ increasing from 0.42% to 1.02%. The persistence of Fe, Mn, and Pb is liked to China’s steel industry, the world’s largest since 1996 [29]. Despite the annual fluctuation in crude steel production (0.9%, −2.3%, 1.2%, 5.7%, 6.6%, 8.3%, 5.2%, and −3% from 2014 to 2021), sustained industrial activity limited reductions in these metals. Steel manufacturing relies on iron ore, manganese ore, and recycled metals, generating Fe-rich particulate emissions [5,30]. Consequently, soils near the mining, smelting, and metallurgical industries through sedimentation typically exhibit elevated levels of Fe, Pb, Zn, and Mn [3,31].

The consistent growth in steel production has led to a relatively smaller decrease in the concentration of Fe, Mn, Zn, and Pb compared with other heavy metals in PM_2.5_. Furthermore, there are significant correlations among these four elements, as evidenced by Spearman correlation analysis (Table 3 and Table 4). Although the correlation analysis for Fe was less satisfactory after 2018 due to the smaller dataset, a significant correlation (r > 0.5) was observed among the top four elements before 2018 and the remaining three elements afterward, suggesting similar sources for Pb, Mn, Zn, and Fe. Studies indicated that the correlation between Fe, Zn, Pb, and Mn is considered a major marker of motor vehicle emissions [14]. Therefore, the small percentage decrease in these heavy metal concentrations may be attributed to the growth in steel production and the number of motor vehicles in China.

**Figure 1:**
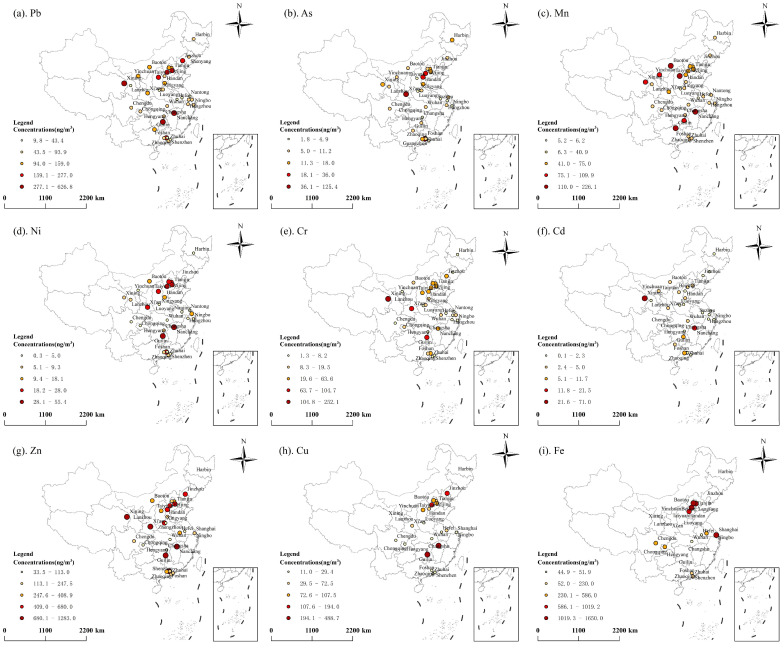
Spatial distribution of the comprehensive concentration of heavy metals in PM_2.5_ before 2018.

**Figure 2:**
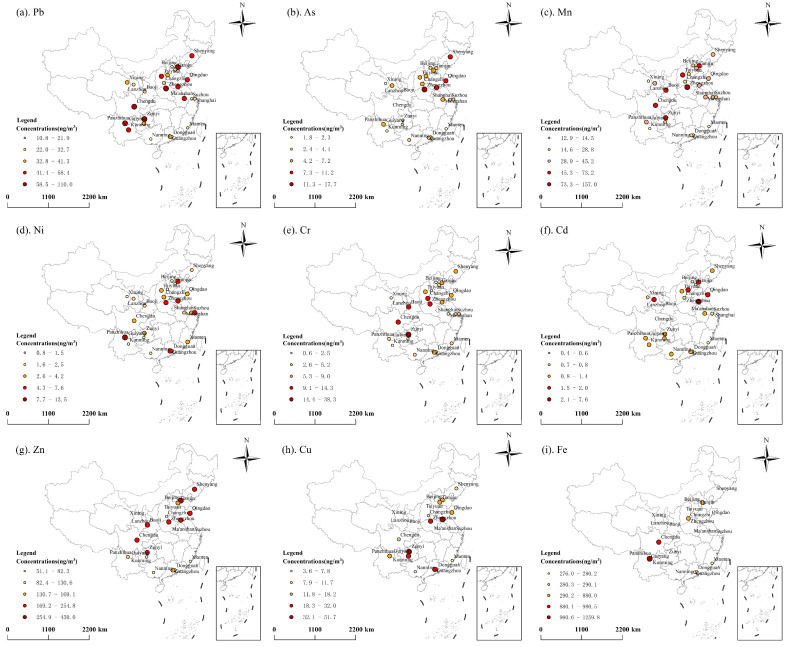
Spatial distribution of the comprehensive concentration of heavy metals in PM_2.5_ after 2018.

To compare the spatial distribution of heavy metals in PM_2.5_ across China before and after the implementation of the policy, 36 major cities and regions before 2018 and 26 major cities and regions after 2018 were divided into six regions (North, Northeast, Northwest, South, East, Southwest China) based on the environmental inspection jurisdiction [32]. The concentrations of heavy metals in these six regions are shown in Figure 1 and Figure 2. Before 2018, the high concentrations of heavy metals clustered in North China, South China, and parts of Northwest China (e.g., Xining and Lanzhou). After 2018, high concentrations persisted in North China and South China, with new hotspots in Southwest China (e.g., Chengdu, Zunyi and Kunming). Before 2018, Cr, Cd, and Cu hotspots occurred in Northwest (e.g., Xining and Lanzhou) and South China (e.g., Hengyang and Changsha). However, after 2018, Cr and Fe became concentrated in Southwest cities (e.g., Chengdu, Panzhihua, Guiyang). A persistent north–south disparity in heavy metals concentrations exists, with northern regions exhibiting higher levels due to industrial density and energy reliance on coal [32,33].

### 3.2. Comparison of PM_2.5_ and Heavy Metal Concentrations Between RICs and GCs Before and After 2018

In RICs, the total PM_2.5_ concentration decreased by 18.7%, from 86.7 μg·m^−3^ before 2018 to 70.5 μg·m^−3^ after 2018 (Table A1). Among the heavy metals in PM_2.5_, Fe exhibited the highest concentration, with average levels of 1279.7 before 2018 and 941.7 ng·m^−3^ after 2018, followed by Zn (595.1 and 264.4 ng·m^−3^), Pb (205.9 and 79.3 ng·m^−3^), Cu (179.8 and 26.6 ng·m^−3^), Mn (95.3 and 47.5 ng·m^−3^), Cr (47.4 and 12.9 ng·m^−3^), As (19.7 and 5.5 ng·m^−3^), Ni (19.9 and 5.0 ng·m^−3^), and Cd (10.1 and 2.1 ng·m^−3^). All nine heavy metal elements showed significant decreasing trends: Cu decreased by 85.2%, Cd by 79.2%, Ni by 74.9%, Cr by 72.8%, As by 72.1%, Pb by 61.5%, Zn by 55.6%, Mn by 50.2%, and Fe by 26.4%. Notably, the proportion of Fe in PM_2.5_ increased from 0.19% before 2018 to 1.40% after 2018, while the proportion of other heavy metals declined. This trend is likely attributed to the stringent regulatory restriction on industrial pollution emissions, particularly in sectors linked to natural resource extraction and processing. However, Fe exhibited a comparatively smaller decline than other metals, potentially due to persistent industrial activity or unique emission sources [34].

**Figure 3:**
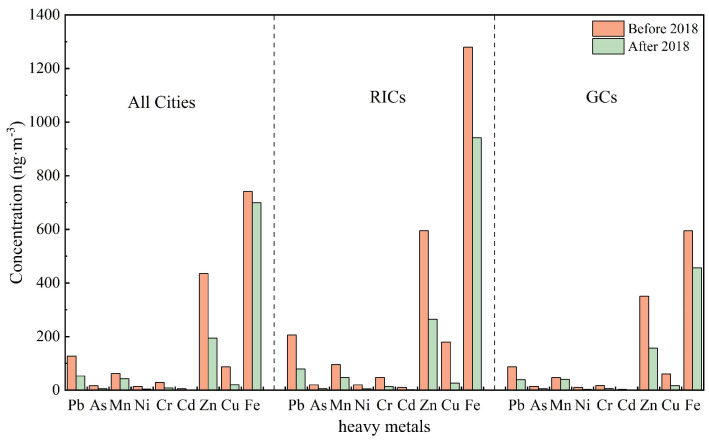
Comparison of the mass concentration of heavy metal elements in atmospheric PM_2.5_ before and after 2018.

In comparison, PM_2.5_ concentrations’ GCs declined by 29.4% from 71.1 μg·m^−3^ pre-2018 to 50.3 μg·m^−3^ post-2018 (Table A1). The average mass concentrations of the heavy metal elements in GCs before 2018 were Fe (594.4 ng·m^−3^), Zn (350.2 ng·m^−3^), Pb (87.6 ng·m^−3^), Cu (60.5 ng·m^−3^), Mn (46.9 ng·m^−3^), Cr (17.0 ng·m^−3^), As (14.2 ng·m^−3^), Ni (9.8 ng·m^−3^), and Cd (2.9 ng·m^−3^) (Figure 3). After 2018, these levels declined for all nine heavy metal elements: Cu by 73.2%, Ni by 71.4%, Cr by 65.9%, Cd by 65.5%, As by 59.9%, Pb by 55.4%, Zn by 55.2%, Fe by 23.2%, and Mn by 14.9%. The proportions of other heavy metals in PM_2.5_, except iron, all decreased after 2018 compared with those before 2018. The proportion of Fe in PM_2.5_ increased from 0.46% before 2018 to 0.91% after 2018. This could be attributed to the accumulation of heavy metals emitted from neighboring industrial areas through long-distance atmospheric transportation [35]. Moreover, the GCs include megacities such as Beijing, Shanghai, Guangzhou, etc., with high population density and vehicular traffic, which, combined with increased coal combustion during the residential heating season, may explain the relatively smaller decline in Mn levels [16,36].

Before and after 2018, atmospheric PM_2.5_ concentrations and atmospheric heavy metal levels varied significantly between RICs and GCs. Prior to 2018, the atmospheric PM_2.5_ concentration in RICs exceeded those in GCs, with an average of 86.7 μg·m^−3^ and 71.1 μg·m^−3^, respectively. Similarly, RICs exhibited a significantly higher concentration ratio of heavy metal in PM_2.5_. Specifically, the average mass concentrations of Cd, Cu, Cr, Pb, Fe, Mn, Ni, Zn, and As in the RICs were 248.3%, 197.2%, 178.8% 135.0%, 115.3%, 103.2%, 103.1%, 69.9%, and 38.7% higher, respectively, compared to GCs (Table A1). Additionally, heavy metals accounted for a higher proportion of PM_2.5_ in RICs, reflecting the region’s industrial structure, which is dominated by mining, ore transportation, and slag accumulation, activities known to emit particulate matter enriched with heavy metals [37]. Additionally, energy extraction and transportation produce substantial soil dust, increasing Mn and Cr levels in RICs [38]. Smelting operations further intensified heavy metals like Pb, Cd, As, and Cr in industrial areas [35]. Therefore, the higher concentrations of PM_2.5_ and heavy metal elements in RICs can be attributed to their energy-intensive and high-pollution activities and elevated demand for resources and energy compared to GCs.

After 2018, the PM_2.5_ concentration in RICs (70.5 μg·m^−3^) remained higher than in GCs (50.3 μg·m^−3^). The average mass concentrations of Cr, Cd, Fe, Pb, Ni, Zn, Cu, and Mn were persistently higher in RICs than in GCs by 122.5%, 110.0%, 106.2%, 102.8%, 78.6%, 68.5%, 64.2%, and 19.0%, respectively. However, GCs exhibited a 3.5% higher As compared to RICs. This anomaly was driven by cities such as Shenyang, Zhengzhou, and Qingdao, where levels exceed 10 ng·m^−3^ (Table A1). As serves as a marker pollutant for coal combustion emissions [36]. In these cities, the sampling points were located in mixed residential and transportation areas, which lacked proximate industrial sources. Consequently, the elevated As levels may instead originate from residential coal heating, vehicular emissions, and long-distance transportation. To improve urban air quality and protect public health, many large cities have relocated coal-burning enterprises to suburbs or small adjacent cities. However, cities like Shenyang, Zhengzhou, and Qingdao remain affected by nearby industrial cities such as Anshan, Luoyang, and Weifang. Wind and atmospheric turbulence can transport mineral dust and industrial fumes to these sampling sites [39]. For instance, Shenyang, although categorized as a GC based on the geographic location of the sampling site, retains industrial influence from equipment manufacturing, internal industrial sources, and coal use in the heating season. Coastal cities like Qingdao and Tianjin also face contributions from ship emissions at nearby ports, which elevated Pb, As, and Cr levels [40,41,42]. Therefore, it can be inferred that the high As level in GCs is related to residential coal combustion, transportation-related emissions, and regional atmospheric dynamics.

### 3.3. Comparison of Variation in Human Health Risk Assessments for Heavy Metals

**Figure 4:**
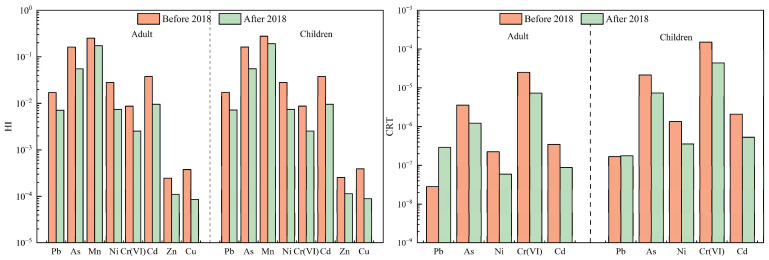
Comparison of HI and CRT of heavy metals in all cities before and after 2018.

**Figure 5:**
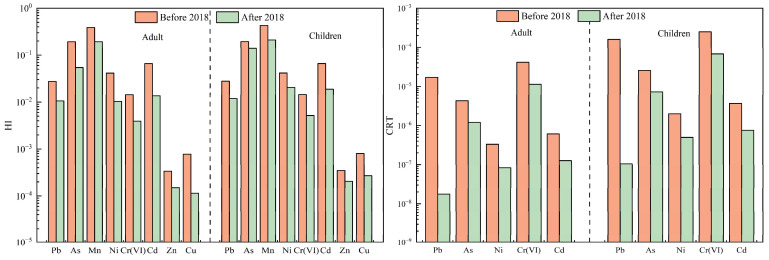
Comparison of HI and CRT of heavy metals in RICs before and after 2018.

**Figure 6:**
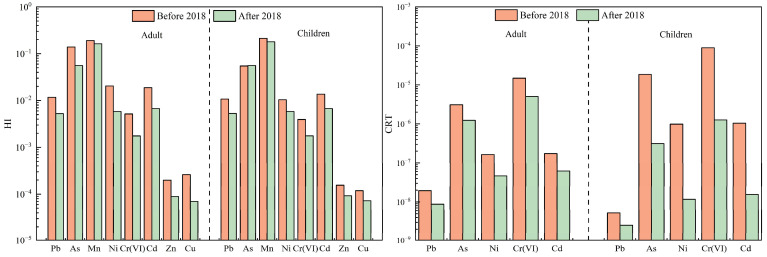
Comparison of HI and CRT of heavy metals in GCs before and after 2018.

Due to their small particle size and large specific surface area, PM_2.5_ exhibits a strong adsorption of harmful substances, including heavy metals, and poses a significant risk to human health. These particles can penetrate deep into the respiratory system, potentially triggering respiratory, immune, and cardiovascular diseases [33]. Heavy metals attached to PM_2.5_ can enter the human body through inhalation, oral ingestion, and dermal contact, accumulating over time and adversely affecting health [13]. Consequently, assessing the health risk associated with atmospheric heavy metals has become a prominent research area. To quantify these risks, this study employed the U.S. EPA health risk evaluation model [25], evaluating combined non-carcinogenic HI and combined CRT for adults and children across all cities, as well as RICs and GCs, before and after 2018 through three exposure pathways: oral ingestion, inhalation, and dermal contact. The HI and CRT of the three uptake pathways are presented in Figure 4, Figure 5 and Figure 6 and Table 5.

For non-carcinogenic risk, the HI values for adults and children in all cities remained below the acceptable threshold (HI = 1) after 2018. However, the HI values for both groups were approximately double before 2018 compared to after 2018, indicating a marked decline in non-carcinogenic risk due to decreased heavy metal concentrations. Carcinogenic risk assessments revealed similar trends; CRT values for adults decreased from 2.9 × 10^−5^ to 9.0 × 10^−6^, while CRT values for children declined from 1.8 × 10^−4^ to 5.2 × 10^−5^. Prior to 2018, the CRT values for children exceeded 1 × 10^−4^, indicating significant carcinogenic risk, whereas adults’ risk fell within the 10^−4^–10^−6^ range, suggesting lower but still notable risk. These findings align with previous studies. For example, the CRT values for As, Cd, Co, Cr(VI), and Ni in Beijing’s PM_2.5_ decreased from 1.08 × 10^−5^ in 2016 to 6.50 × 10^−6^ in 2021–2022 [43]. Similarly, Wang et al., and Zhao et al. revealed that the carcinogenic risks of Cd, Pb and Ni in PM_2.5_ in Tianjin ranged between 10^−6^ and 10^−4^ before 2018 but dropped below 10^−6^ after 2018 [27,44]. Collectively, these results demonstrate a consistent decline in both HI and CRT for both adults and children after 2018. This reduction correlates with the implementation of the policy, which strengthened emission control and pollution mitigation measurements. The policy intends to reduce industrial emissions, improve energy efficiency, and regulate coal combustion, effectively mitigating heavy metal concentrations in PM_2.5_ and thereby lowering the associated health risks posed by atmospheric heavy metals.

In RICs, the HI values of adults and children remained below acceptable levels both before and after 2018, with CRT values decreasing from 6.4 × 10^−5^ to 1.3 × 10^−5^ for adults and from 4.4 × 10^−4^ to 7.6 × 10^−5^ for children, respectively. GCs followed a similar pattern: adult CRT values decreased from 1.8 × 10^−5^ to 1.6 × 10^−6^, and children’s CRT values dropped from 1.1 × 10^−4^ to 6.4 × 10^−6^. HI was 1.5–2.0 times higher in RICs than in GCs before and after 2018. Across both city types, HI and CRT values declined after 2018, with CRT decreasing by 69.0–71.1%, aligning with reduced heavy metal levels, yet children consistently faced a higher risk than adults, likely due to physiological vulnerability and behavioral factors (e.g., higher inhalation rates per body weight). Residents of RICs also face elevated health risks compared to GCs, aligning with Li et al.’s finding that the cancer risk levels correlate with heavy metal concentrations, particularly in industrial zones [32].

Mn and As posed the highest non-carcinogenic risk levels among all heavy metals throughout the whole study period, while As and Cr(VI) dominated carcinogenic risk. The contributions of As and Cr(VI) to the CRT were 11.7–14.0% and 81.1–86.2%, respectively. The concentrations of As (16.4 ng·m^−3^) and Cr(VI) (4.1 ng·m^−3^) before 2018 exceeded the standard limits (6 ng·m^−3^ for As, 0.025 ng·m^−3^ for Cr(VI)) set by the China Ambient Air Quality Standard (GB 3095-2012) [45], with exceedance rates of 78% for As and 97% for Cr(VI), respectively. Even after 2018, the exceedance rates remained at 17% and 84%, respectively. The exceedances of As and Cr(VI) in the RICs were greater than in the GCs, as were the carcinogenic risk values for As and Cr(VI). These trends mirror findings by Yu et al. [20], underscoring the persistent threat of these metals. The sustained exceedance of As and Cr(VI) thresholds, even after regulatory interventions, highlights the need for stricter emission controls on high-risk carcinogens, particularly in RICs. Prioritizing industrial source regulation, enhancing air quality monitoring, and addressing transboundary pollution are critical to mitigating health risks.

## 4. Conclusions

The implementation of the Three-Year Action Plan to Win the Blue Sky Defense War has significantly reduced atmospheric PM_2.5_ and associated heavy metals concentrations (Pb, As, Mn, Ni, Cr, Cd, Zn, Cu, and Fe), underscoring the policy’s success in improving air quality. However, correlation analyses show that metals linked to industrial and vehicular emission, such as Fe, Mn, Zn, and Pb, exhibited smaller reductions compared to others, likely due to rising steel production and motor vehicle usage. Geographically, heavy metals hotspots before 2018 were concentrated in northwest, northern, and southern China. Post-2018, these high-concentrations areas shifted southwestward while remaining prevalent in northern and southern regions. Although both RICs and GCs exhibited declinations in heavy metal levels after 2018, the reductions were more pronounced in RICs.

The health risk assessment demonstrated that HI remained within acceptable thresholds (HI < 1) for all populations throughout the whole study period. CRT, however, posed greater concern: pre-2018 CRT values for adults fell within the 10^−6^–10^−4^ range (indicating potential risk), while the CRT for children exceeded 10^−4^ (definitive risk). These risks were consistently higher in RICs than in GCs, aligning with regional disparities in industrial activity. Post-2018 declines in HI and CRT paralleled reductions in heavy metal concentrations, affirming the policy’s effectiveness in safeguarding public health.

However the main carcinogens, As and Cr(VI), persist as critical threats, with pre-2018 concentrations exceeding China standards by 78% (As) and 97% (Cr(VI)). Post-2018 exceedance rates remained elevated at 17% and 84%, respectively, particularly in RICs. These findings underscore the urgency of controls on industrial and combustion-related emissions of high-risk carcinogens. This study provides a scientific foundation for refining air quality policies, emphasizing targeted emission regulations, enhancing monitoring in high-risk regions, and employing adaptive strategies to mitigate residual health threats. Sustained efforts to curb toxic pollutants are essential to consolidating gains from the Three-Year Action Plan to Win the Blue Sky Defense War and ensuring long-term environmental and public health resilience.

## Figures and Tables

**Table 1:** Parameters and their values entered in the health risk assessment model.

Argument	Definition	Unit	Numerical Value	
			Children	Adult
ABS	Skin absorption factor		0.03 (As), 0.1 (Pb), 0.001 (Cd), 0.01 (else)	
AF	Skin adhesion factor	mg·cm^−2^	0.2	0.07
AT	Mean life	d	ED $\;\times \;365\left(noncarcinogenic\;effect\right)$ 70 × 365 (carcinogenesis)	ED $\;\times \;365\left(noncarcinogenic\;effect\right)$ 70 × 365 (carcinogenesis)
AT_n_	Mean life	h	ED $\;\times 365\;\times \;24\left(noncarcinogenic\;effect\right)$ 70 × 365 × 24 (carcinogenesis)	ED $\;\times \;365\;\times \;24\left(noncarcinogenic\;effect\right)$ 70 × 365 × 24 (carcinogenesis)
BW	Per capita weight	kg	15	70
CF	Conversion factor	kg·mg^−1^	1.0 × 10^−6^	1.0 × 10^−6^
ED	Exposure period	a	6	24
EF	Exposure frequency	d·a^−1^	180	180
ET	Exposure time	h·d^−1^	24	24
IngR	Hand–mouth intake	mg·d^−1^	200	100
SA	Skin surface area	cm^2^	2800	5700

**Table 2:** Reference doses of heavy metals.

Types of Heavy Metals	RfD_0_	RfC_i_	GIABS	SF_0_	IUR
Pb	3.50 × 10^−3^	3.52 × 10^−3^	1	0.0085	0.000012
As	3.00 × 10^−4^	1.50 × 10^−5^	1	1.5	0.0043
Mn	1.43 × 10^−5^	0.50 × 10^−4^	1	—	—
Ni	2.00 × 10^−2^	9.00 × 10^−5^	0.04	—	0.00026
Cr	3.00 × 10^−3^	1.00 × 10^−4^	0.025	0.5	0.084
Cd	1.00 × 10^−3^	1.50 × 10^−5^	0.025	—	0.0018
Zn	3.00 × 10^−1^	3.01 × 10^−1^	1	—	—
Cu	4.00 × 10^−2^	4.02 × 10^−2^	1	—	—

**Table 3:** Correlation analysis results of heavy metal elements before 2018.

Item	Pb	As	Mn	Ni	Cr	Cd	Zn	Cu	Fe
Pb	1								
As	0.532 **	1							
Mn	**0** .812 **	0.372	1						
Ni	0.742 **	0.359	0.625 **	1					
Cr	0.804 **	0.487 *	0.652 **	0.842 **	1				
Cd	0.783 **	0.602 **	0.767 **	0.678 **	0.633 **	1			
Zn	**0** .767 **	0.663 **	**0** .749 **	0.728 **	0.761 **	0.597 **	1		
Cu	0.835 **	0.262	0.758 **	0.917 **	0.729 **	0.633 *	0.701 **	1	
Fe	**0** .669 *	0.595	**0** .866 **	0.689 *	0.845 **	0.300	**0** **.508**	0.761 **	1

Note: The black bold letters in the table show the correlation coefficients between Fe, Mn, Zn, and Pb. * indicates *p* < 0.05; ** indicates *p* < 0.01; data with * and ** indicate significant correlation.

**Table 4:** Correlation analysis results of heavy metal elements after 2018.

Item	Pb	As	Mn	Ni	Cr	Cd	Zn	Cu	Fe
Pb	1								
As	0.412 *	1							
Mn	**0** .630 **	0.636 **	1						
Ni	0.446 *	0.482 *	0.498 *	1					
Cr	0.476 *	0.351	0.700 **	0.706 **	1				
Cd	0.678 **	0.421	0.676 **	0.347	0.396	1			
Zn	**0** .657 **	0.450	**0** .852 **	0.414	0.627 **	0.739 **	1		
Cu	0.379	−0.011	0.340	0.315	0.168	0.543 *	0.338	1	
Fe	**0** **.524**	0.607	**0** **.611**	0.333	0.167	0.468	0.286	0.071	1

Note: The black bold letters in the table show the correlation coefficients between Fe, Mn, Zn, and Pb. * indicates *p* < 0.05; ** indicates *p* < 0.01; data with * and ** indicate significant correlation.

**Table 5:** Heavy metal CRT and HI values before and after 2018.

Type of City	Time	Age	HI	CRT
All Cities	Pre-2018	Adults	0.5	2.9 × 10^−5^
		Children	0.5	1.8 × 10^−4^
	Post-2018	Adults	0.3	9.0 × 10^−6^
		Children	0.3	5.2 × 10^−5^
RICs	Pre-2018	Adults	0.7	6.4 × 10^−5^
		Children	0.8	4.4 × 10^−4^
	Post-2018	Adults	0.3	1.3 × 10^−5^
		Children	0.3	7.6 × 10^−5^
GCs	Pre-2018	Adults	0.4	1.8 × 10^−5^
		Children	0.4	1.1 × 10^−4^
	Post-2018	Adults	0.2	1.6 × 10^−6^
		Children	0.2	6.4 × 10^−6^

## Data Availability

No new data were created or analyzed in this study. Data sharing is not applicable to this article.

## Appendix A

**Table A1:** PM_2.5_ and heavy metal concentrations (ng·m^−3^) in All Cities, RICs, and GCs before and after 2018 in China.

Year Range	City	Type of City	PM_2.5_	Pb	As	Mn	Ni	Cr	Cd	Zn	Cu	Fe	Data Source
2014–2018	Hengyang	RICs	111,180	456.9	8.4	109.9	55.4	104.7	11.7	898.8	488.7	-	[46]
	Baotou		53,400	143.8	8.5	211.4	16.6	19.5	2.5	280.9	-	-	[47]
	Handan		91,180	115	13.5	28.3	18.1	12.6	4.9	187.8	-	-	[48]
	Suzhou		66,200	76.2	4.1	-	-	6.9	1.5	-	-	-	[49]
	Ningbo		84,000	61.9	4.9	36.8	4.1	3.4	1.5	-	-	-	[34]
	Foshan		47,000	277	91.6	-	28	36	4.4	1283	-	-	[47]
	Zhaoqing		39,375	93.9	17.6	-	8.8	10.2	10.2	408.9	-	-	[47]
	Baodinng		180,000	235	30	55	27.5	32.5	5	622.5	194	1340	[50]
	Taiyuan		97,300	241.3	1.8	171.9	25.3	63.6	2.8	338.2	83.1	-	[51]
	Xining		49,688	626.8	18	97.7	9.3	252.1	70.9	958.9		-	[47]
	Shijiazhuang		99,475	84.3	35.9	71.4	-	31.4	1.9	634.7	60.5	1019.2	[52]
	Yinchuan		75,000	124.1	9.2	96.1	1.2	2.9	3.3	-	-	-	[53]
	Langfang		133,000	140	12.5	75	25	40	-	337.5	72.5	1480	[54]
	Luoyang	GCs	72,236	9.8	3.1	6.2	0.3	1.3	0.1	-	11	-	[55]
	Xingyang		78,310	60	4.5	-	-	10	-	510	-	-	[56]
	Jinzhou		41,250	225.5	-	-	-	49	0.7	679.8	158.5	-	[57]
	Guangzhou		37,000	40	8.6	25	3.2	-	1.4	190	22	230	[7]
	Zhuhai		45,000	59	-	28	7	6	-	149	20	212	[58]
	Shenzhen		39,100	25.6	4.9	19.5	4.2	2.3	0.8	163.3	29.4	-	[59]
	Hangzhou		92,000	78.8	8.1	34	2.5	3.1	1.9	-	-	-	[34]
	Guilin		66,000	102	11.2	173	-	-	4.9	-	-	-	[60]
	Xi’an		50,600	159	125.4	61.5	21.5	100.9	3.3	1264.8	43.4	-	[61]
	Nanchang		29,740	468.7	-	226.1	51.2	32	21.5	1141.1	343.4	-	[62]
	Beijing		126,000	120	10	55	25	32.5	-	247.5	107.5	1650	[34]
	Tianjin		133,000	190	13.3	60	23.3	36.7	3.3	680	100	-	[34]
	Shanghai		94,600	69.7	-	-	14.9	16.9	-	215	24.2	1340	[63]
	Chongqing		60,695	50.4	-	37.7	4.2	11.1	-	113	11.3	586	[1]
	Shenyang		76,225	62.3	10.5	28.3	-	8.2	1.3	-	-	-	[64]
	Harbin		93,250	57.3	14.7	25.4	2.8	3.8	0.9	-	-	-	[65]
	Nanjing		39,250	23.5	2	20.8	4.9	6.7	1	99	50.9	455.2	[66]
	Zhengzhou		165,200	57.7	8.2	40.9	-	-	2.2	72.7	-	-	[67]
	Changsha		46,900	55.8	3.8	18.8	7.6	5.5	0.5	33.5	-	-	[46]
	Chengdu		64,184	55.4	10.8	33.8	2.1	5.6	-	238	18.7	456	[1]
	Nantong		58,400	28.3	6.7	40.6	4.7	2.5	1	-	-	-	[68]
	Hefei		81,000	12.6	-	5.2	-	10	-	273.5	11.3	44.9	[26]
	Lanzhou		50,699	43.4	8.4	40.5	6.6	-	2.3	-	-	-	[69]
	Wuhan		57,027	90.9	8.2	25.9	3.73	-	3	67.7	15.5	51.9	[45]
Avg. ± std		RICs	86,676.8 ± 39,372.6	205.9 ± 167.2	19.7 ± 23.8	95.3 ± 57.8	19.9 ± 15.1	47.4 ± 67.7	10.1 ± 19.5	595.1 ± 354.6	179.8 ± 180.8	1279.7 ± 236.2	
		GCs	71,155.2 ± 33,133.5	87.6 ± 93.3	14.2 ± 27.1	46.9 ± 50.9	9.8 ± 12.2	17.0 ± 23.3	2.9 ± 4.8	350.2 ± 353.5	60.5 ± 83.7	594.4 ± 544.2	
		All Cities	76,329.1 ± 35,591.3	127.0 ± 133.2	16.4 ± 25.0	61.6 ± 56.8	13.4 ± 14.0	28.6 ± 47.1	5.7 ± 13.0	434.7 ± 367.1	87.6 ± 119.1	741.2 ± 567.0	
2019–2021	Tangshan	RICs	97,500	110	4.1	60	5.3	8.6	1.9	450	17.7	880	[70]
	Zaozhuang		91,000	46.4	9.4	45.2	6.8	6.5	7.6	215	51.7	-	[71]
	Panzhihua		33,000	82.4	7.2	34.8	13.5	5.1	1.2	130.6	18	1259.8	[72]
	Zunyi		47,600	78.5	2.1	64.4	2.9	38.3	1.2	213.3	49.5	-	[73]
	Taiyuan		87,130	57.6	5.4	51.7	3.1	6.9	1	-	-	-	[74]
	Xining		34,331	36	2.1	12.9	1	1.6	0.7	-	-	-	[75]
	Caofeidian		89,680	230.4	9.3	107.8	7.1	32.7	2.8	495.4	14.7	762.3	[76]
	Changzhi		56,100	30.8	4.9	21.5	4.2	14.3	0.7	82.3	7.8	864.5	[22]
	Shijiazhuang		98,130	41.3	5.4	28.8	1.1	2.5	1.6	-	-	-	[77]
	Maanshan	GCs	51,750	50.1	6.2	35.3	1.8	1.7	1.4	-	-	-	[78]
	Baoji		51,500	27.5	-	73.2	2.5	13.4	-	234.8	-	-	[79]
	Dongguan		35,800	17.9	4.7	19	1.9	4.6	0.7	109.8	11.7	290.1	[80]
	Guangzhou		40,300	37	3.1	20	5.8	9	1.1	161	32	276	[81]
	Suzhou		46,760	21.9	3.5	30.5	3.2	4.6	0.7	-	-	-	[82]
	Beijing		48,000	21.7	2.8	21.1	0.9	0.6	0.5	-	-	-	[83]
	Tianjin		59,000	27.1	6.9	34.3	1.5	3.3	0.6	169.1	18.2		[83]
	Shanghai		40,610	32.7	3.3	22.3	5	5.2	-	-	-	-	[84]
	Shenyang		85,100	58.4	11.2	36.1	2.1	6.6	1	206.4	9.4		[85]
	Zhengzhou		52,000	100	17.7	157	7.6	11.7	-	209.8	29.2	-	[86]
	Chengdu		115,300	73.7	-	60	3.8	11.1	-	254.8	11	980.5	[39]
	Kunming		26,670	48.9	1.8	13.4	0.8	1.9	1.1		-	-	[87]
	Guiyang		26,750	37.8	2.1	19.1	0.8	1.8	1.1	51.1	25.1	-	[88]
	Qingdao		85,500	52	10.8	42	3	8	1.8	211.1	14.6	-	[44]
	Nanning		34,000	19.8	3.2	-	1.3	3.7	1	64.2	3.6	-	[89]
	Lanzhou		34,514	27.9	5.5	41.7	2	-	2	-	-	-	[68]
	Xiamen		21,620	10.8	2.3	14.5	3.6	5.1	0.4	53.4	7.3	280.2	[40]
Avg. ± std		RICs	70,496.8 ± 27,393.7	79.3 ± 62.3	5.5 ± 2.7	47.5 ± 28.5	5.0 ± 3.9	12.9 ± 13.4	2.1 ± 2.2	264.4 ± 169.7	26.6 ± 19	941.7 ± 218.4	
decline range			18.7%	61.5%	72.1%	50.2%	74.9%	72.8%	79.2%	55.6%	85.2%	26.4%	
Avg. ± std		GCs	50,304.4 ± 24,532.6	39.1 ± 22.8	5.7 ± 4.4	39.9 ± 35.3	2.8 ± 1.0	5.8 ± 3.9	1.0 ± 0.5	156.9 ± 75.3	16.2 ± 9.6	456.7 ± 349.3	
decline range			29.4%	55.4%	59.9%	14.9%	71.4%	65.9%	65.5%	55.2%	73.2%	23.2%	
Avg. ± std		All Cities	57,294 ± 26,856.8	53 ± 44.2	5.6 ± 3.8	42.7 ± 32.6	3.6 ± 2.9	8.4 ± 9.0	1.5 ± 1.5	194.8 ± 123.9	20.1 ± 14.2	699.2 ± 374.1	
decline range			24.9%	58.3%	65.9%	30.7%	73.1%	70.6%	73.7%	55.2%	77.1%	5.7%	
